# Top dusted adhesive tape sample preparation method for the X-ray diffraction analysis of small powder sample volumes with the Bragg–Brentano setup

**DOI:** 10.1107/S1600576724011920

**Published:** 2025-02-01

**Authors:** Daniel Dickes, Uwe Glatzel

**Affiliations:** a Metals and Alloys University of Bayreuth Professor-Rüdiger-Bormann-Straße 1 Bayreuth95447 Germany; Wilfrid Laurier University, Waterloo, Ontario, Canada

**Keywords:** X-ray diffraction, XRD, powder diffraction, sample preparation, adhesive tapes, small powder volumes

## Abstract

When performing X-ray diffraction (XRD) analysis of small powder sample volumes with the Bragg–Brentano setup, choosing an appropriate sample preparation method is important. This work provides practical information on the conventional top dusted sample preparation method and the top dusted adhesive tape sample preparation method. In the latter, the powder of interest is dusted onto an adhesive tape. Because adhesive tapes contribute to the measured XRD signal as background noise, care must be taken when using a particular adhesive tape. This work assists XRD users in selecting the most suitable adhesive tape for their purpose by providing the diffractograms (Cu *K*α_1_ radiation) of 13 commercially available adhesive tapes used for office and packing applications. In general, tapes with a matte acetate or polyvinyl chloride backing are recommended as they cause a comparatively low background signal without high-intensity reflections.

## Abstract 2

This work provides practical information on the conventional top dusted sample preparation method and the top dusted adhesive tape sample preparation method. For the top dusted adhesive tape method, tapes with matte acetate or polyvinyl chloride backing are recommended as they cause a comparatively low background signal without high-intensity reflections.

## 1. Introduction

X-ray diffraction (XRD) analysis of powder samples is a widely used and established method in materials research when it comes to the atomic scale structural characterization of materials (Waseda *et al.*, 2011 ▸). Although numerous textbooks cover the fundamental principles of XRD, the experimental instrumentations and setups for XRD analysis and the evaluation of diffraction data [*e.g.* Klug & Alexander (1976 ▸), David (2006 ▸) and Dinnebier (2009 ▸)], practical sample preparation techniques are less thoroughly addressed (Buhrke *et al.*, 1999 ▸). The textbook by Buhrke *et al.* (1999 ▸) is a notable exception, presenting various sample preparation techniques with a practical approach. At the same time, it provides sufficient background information on technique-specific use cases.

**Figure 1:**
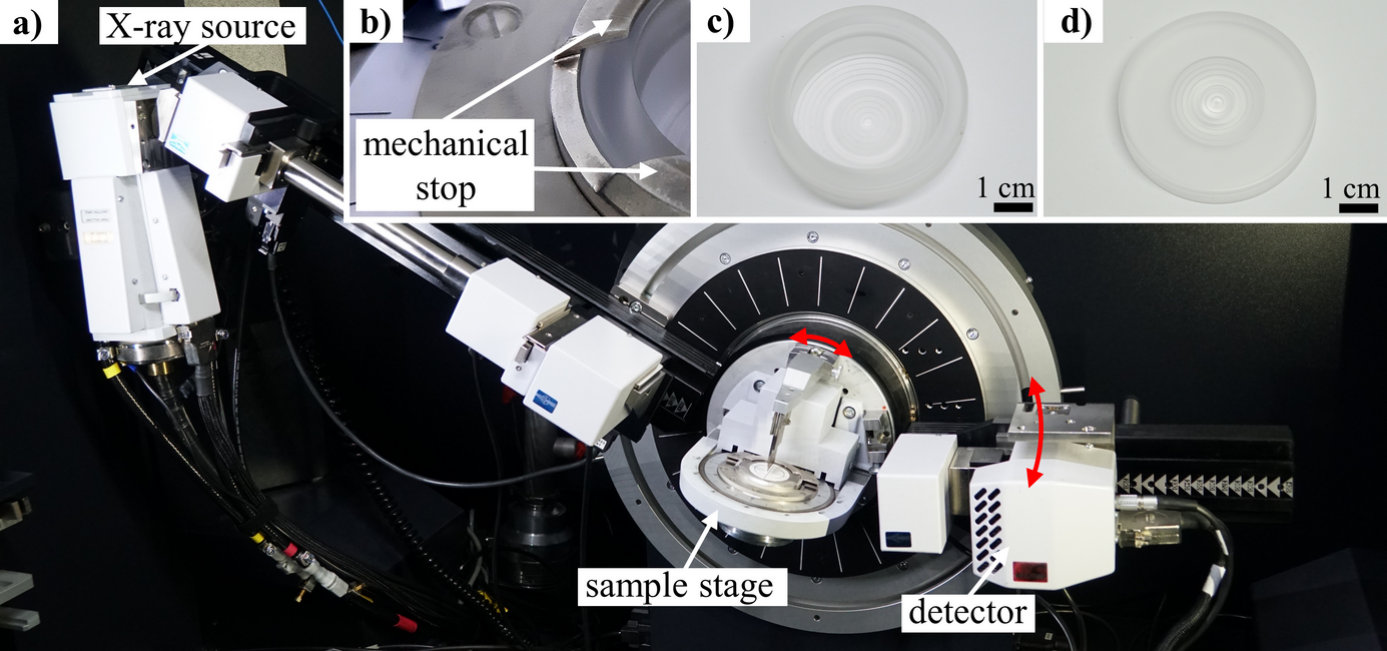
(*a*) Bruker D8 Discover diffractometer in a Bragg–Brentano setup with a rotation sample stage, (*b*) a magnified portion of the sample stage, (*c*) a suitable deep-well sample holder and (*d*) a front-loading cavity sample holder.

According to Buhrke *et al.* (1999 ▸), most commercial powder diffraction systems use the Bragg–Brentano setup. Different implementations of the Bragg–Brentano setup exist, along with a variety of sample stages. Fig. 1 ▸(*a*) shows one implementation of such a Bragg–Brentano setup, realized with a Bruker D8 Discover diffractometer. In this specific setup, a tiltable rotation sample stage is mounted at the centre of the goniometer. This sample stage allows for an easy exchange of samples, as the samples are prepared in sample holders and then placed on the stage at a geometrically defined fixed position. The defined position is ensured through a system in which a spring pushes the sample holder against a mechanical stop [Fig. 1 ▸(*b*)]. As no height correction is possible with this sample stage, accurate positioning of the sample in the sample holder must be ensured by aligning the sample surface with the top of the specimen holder as the reference surface.

Depending on the nature of the sample and the analysis task being undertaken, different sample preparation methods are available, each requiring different sample holders. Figs. 1 ▸(*c*) and 1 ▸(*d*) show typical sample holder types available for the Bruker D8 sample stage. The deep-well holder [Fig. 1 ▸(*c*)] is used in combination with modelling clay to mount solid samples, *e.g.* polycrystalline bulk metals, which, due to their polycrystalline nature, are regarded as quasi-powders. The front-loading cavity holder [Fig. 1 ▸(*d*)] is used for powders and is commonly deployed when correct relative intensities are not important (Buhrke *et al.*, 1999 ▸). Such sample holders are typically made from an amorphous material like polymethyl methacrylate (PMMA), as in this case, or from a crystalline material like steel or aluminium. The simplicity of PMMA front-loading cavity sample holders results in a comparatively low price of approximately EUR 70 per piece (Bruker Corporation, https://store.bruker.com/en-DE/collections/sample-holders-and-preparation) and no further operating costs as they are reusable.

**Figure 2:**
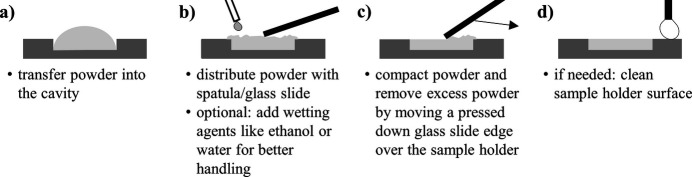
Schematic representation of the sample preparation routine with front-loading cavity sample holders.

Fig. 2 ▸ visualizes the preparation routine associated with front-loaded sample holders. The preparation is straight­forward and does not require sophisticated equipment.

**Figure 3:**
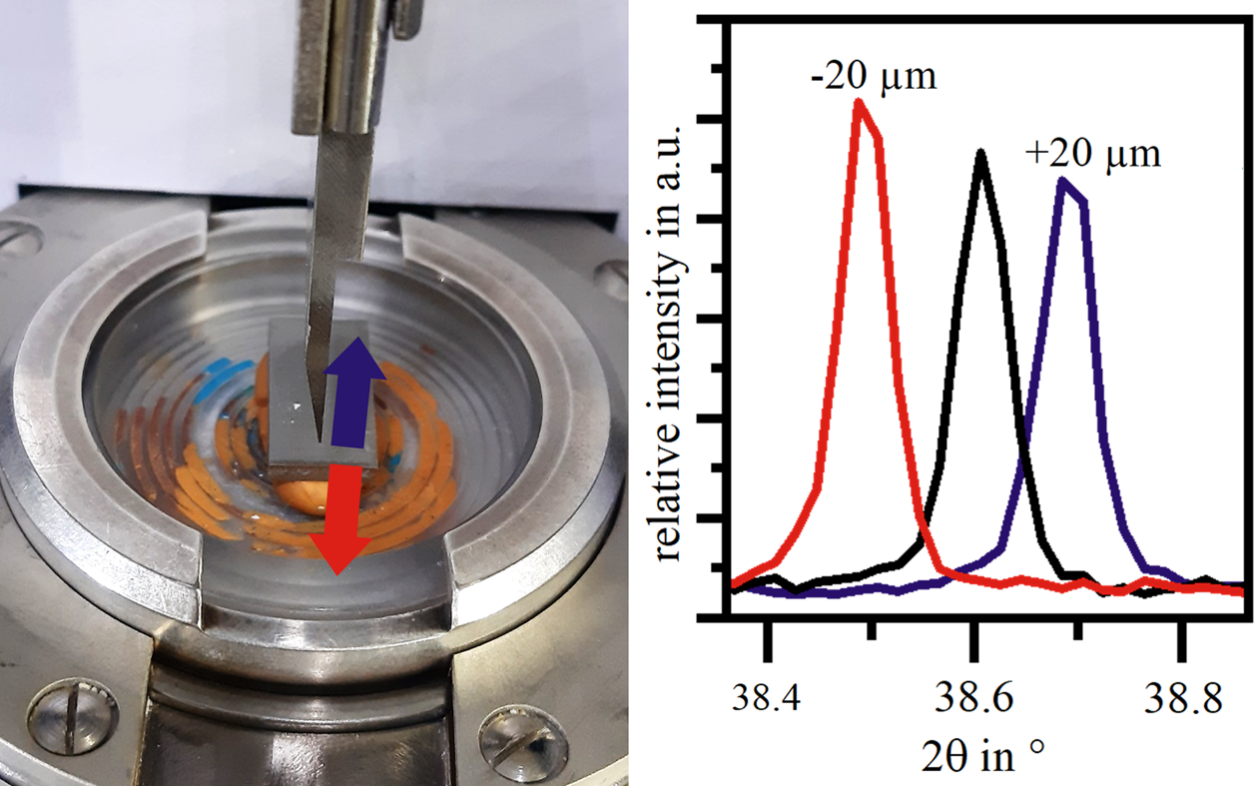
Illustration of positive and negative sample offsets and their effect on the diffraction peak position.

However, in everyday situations, the amount of powder available for an investigation may be insufficient to completely fill the cavity of a standard front-loading cavity sample holder. This causes two problems if a setup like the one presented in Fig. 1 ▸, with a tiltable sample stage and the absence of a height correction mechanism, is used. Firstly, the powder will be insufficiently compacted in the cavity, which is itself a source of inaccuracies, and it may move or even fall out of the cavity during the measurement when the sample stage is tilted. Secondly, an offset occurs between the ideal sample surface position, being in plane with the sample holder reference surface, and the actual sample surface position. Such an offset causes a diffraction peak position shift in the corresponding diffractogram. An offset of ∼25 µm is associated with a diffraction peak position shift of 0.01° 2θ (Buhrke *et al.*, 1999 ▸). Fig. 3 ▸ shows a bulk titanium alloy sample analysed in the ideal position and with 20 µm positive and negative offsets. The corresponding diffractograms clearly reveal the effect of the offsets on the diffraction peak position.

One option to avoid sample offsets due to an insufficiently filled cavity is to manufacture sample holders with smaller cavities tailored to the available amount of powder. This can be easily done with a milling machine if the sample holders are made from PMMA or metal. It has to be considered that such smaller cavities may lead to a noticeable contribution of the sample holder material to the measured signal, resulting in an increase in background. This happens once the cavity volume, and hence the volume of the powder sample, becomes smaller than the total X-ray irradiated volume.

**Figure 4:**
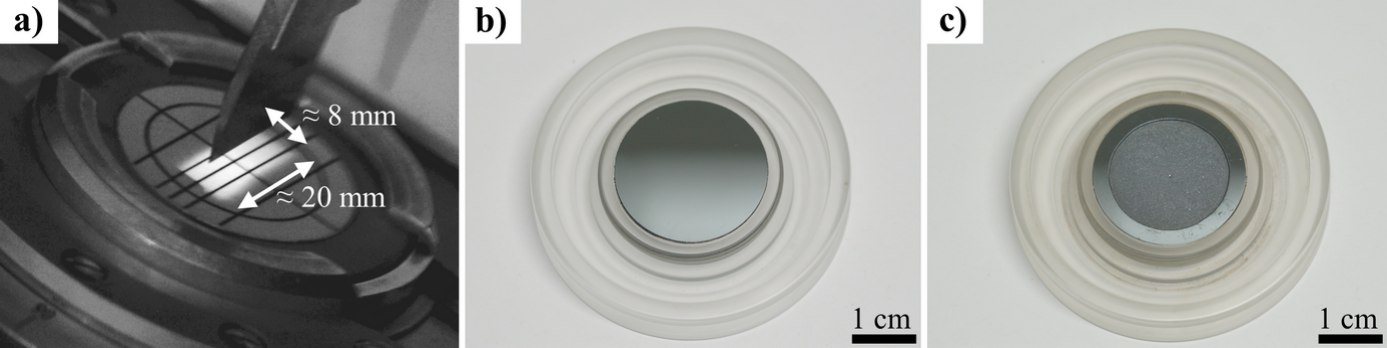
(*a*) X-ray illuminated area visualized with a fluorescence screen, (*b*) zero-background plate and (*c*) zero-background plate with a cavity.

Fig. 4 ▸(*a*) shows the X-ray irradiated area for the Bragg–Brentano setup shown in Fig. 1 ▸(*a*) (θ = 5°, divergence slit mode, opening degree = 0.2°) with the help of a fluorescent screen. For this setup, cavities of diameters less than 20 mm lead to irradiation of the sample holder and, therefore, a contribution of the sample holder material to the measured diffraction signal. To minimize this effect, the X-ray irradiated area can be reduced by adjusting the X-ray optics, provided that suitable optical components are available and they fit into the setup. This may not always be the case. Alternatively, ‘zero-background’ specimen holders [Fig. 4 ▸(*b*)] are available. They consist of a single crystal, *e.g.* silicon, oriented in such a way that no disturbing diffraction pattern is produced (Narasimha Rao *et al.*, 1996 ▸). While zero-background specimen holders with cavities exist [Fig. 4 ▸(*c*)], tailoring the cavity volumes to ensure adequate filling in the case of small powder volumes is costly. The price of such a specimen holder is approximately EUR 350 (KS Analytical Systems, https://store.ksanalytical.com/products/zero-background-sample-holders-with-well) and thus around five times more expensive than a PMMA specimen holder.

The top dusted method is a suitable approach to avoid the offset problem occurring with small powder volumes in combination with front-loading cavity sample holders. This method is also useful when preferred orientation effects must be avoided (Buhrke *et al.*, 1999 ▸).

**Figure 5:**
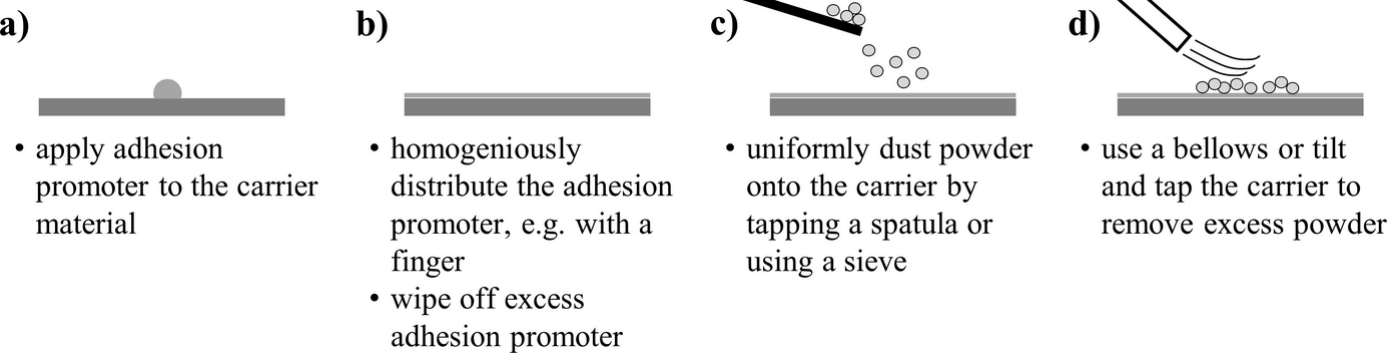
Schematic representation of the powder specimen preparation routine with the top dusted method.

Fig. 5 ▸ visualizes the preparation steps of the top dusted method. An adhesion promoter is applied and homogeneously distributed on a carrier material. The powder of interest is then dusted onto the carrier either by tapping a spatula loaded with the powder or by using a sieve, as recommended by Buhrke *et al.* (1999 ▸). Excess powder is removed with the help of a bellows or by tilting and tapping the carrier.

**Figure 6:**
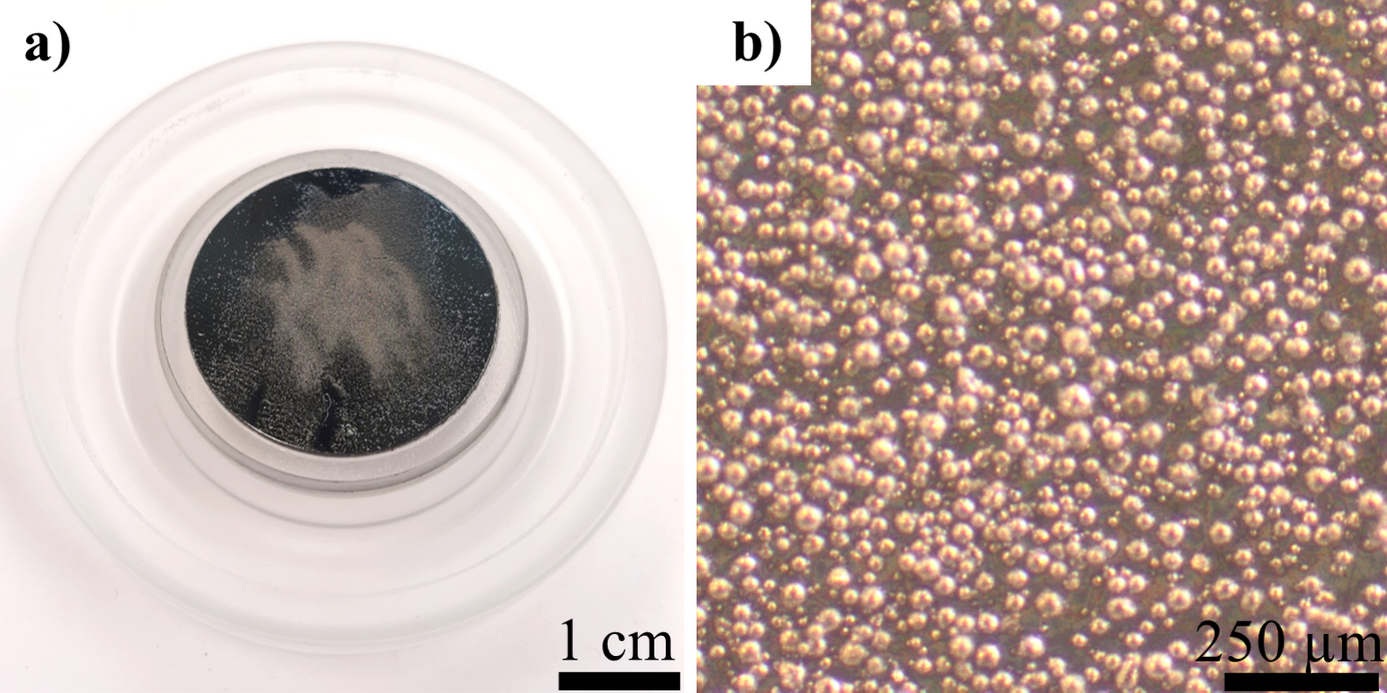
(*a*) Zero-background sample holder with petroleum jelly and dusted Ti6Al4V powder and (*b*) corresponding optical microscopy image of the powder.

Fig. 6 ▸(*a*) shows a Ti6Al4V powder sample prepared according to the steps given in Fig. 5 ▸. In this case, the carrier material is a flat zero-background specimen holder. The adhesion promoter is petroleum jelly. Fig. 6 ▸(*b*) provides an optical microscopy image of the dusted powder, confirming that an adherent single-particle layer is achieved with this method. No significant offset from the ideal sample surface position exists. However, the X-ray irradiated volume does not only cover the powder particles. The X-rays also target the carrier material and the adhesion promoter, potentially contributing to the measured signal in the form of background, with background being defined as all intensity not directly caused by crystalline diffraction of the investigated sample (Buhrke *et al.*, 1999 ▸). Zero-background sample holders, or equivalent silicon wafers, are a good choice to minimize the contribution of the carrier material to the measured signal. Alternatively, microscope glass slides can be used (Buhrke *et al.*, 1999 ▸). As adhesion promoter, Buhrke *et al.* (1999 ▸) recommend pure petroleum jelly (Vaseline) without further ingredients. Depending on the amount of petroleum jelly used, an amorphous reflection in the measured signal has to be considered. In some scenarios, dispersing the powder sample in acetone on a zero-background plate may be an alternative.

The top dusted tape method is a variation of the top dusted method. Instead of a carrier material and an adhesion promoter, commercial adhesive tapes are used. They combine the functions of the carrier material and the adhesion promoter. According to Buhrke *et al.* (1999 ▸), ‘Most transparent mending tapes are usable but blank runs need to be made to ascertain any potential interfering lines.’ A huge number of different adhesive tapes are commercially available, with different intended uses. They differ in their backing material, their type of adhesive, and the thickness of the backing and adhesive layer. Consequently, the diffraction behaviour differs, which has been underlined in XRD experiments on polymer films comparing Mylar (stretched polyethyl­ene terephthalate, PET) and polyether ether ketone (PEEK; Aparicio *et al.*, 2024 ▸). This different diffraction behaviour results in a tape-specific contribution to the measured signal when used for the top dusted adhesive tape method. The backing material and adhesive type are often specified in technical data sheets, while more detailed information, *e.g.* on the degree of crystallinity or a preferred orientation, is usually not available. A prediction of the diffraction behaviour of an adhesive tape based solely on technical data sheets is therefore not possible. Only an experimental XRD analysis can reveal the suitability of an adhesive tape for the top dusted tape method. Therefore, this work assesses the diffraction behaviour of selected commercially available adhesive tapes used for office and packing applications.

## 2. Experimental

Thirteen commercially available adhesive tapes used for office and packing applications were investigated. The adhesive tapes originate from either 3M Corporation (brand name ‘Scotch’) or tesa SE (brand name ‘tesa’) and were chosen randomly. Table 1 ▸ lists the investigated tapes, providing necessary information to identify the tested products uniquely by a sample ID and their brand name, product name, company product number (BNR for tesa products and 3M ID for 3M products) and international article product number EAN. The backing material, adhesive type and thickness of the tested tapes are provided as given by the manufacturers in the product specification, as well as information on whether the tape is single or double sided.

**Figure 7:**
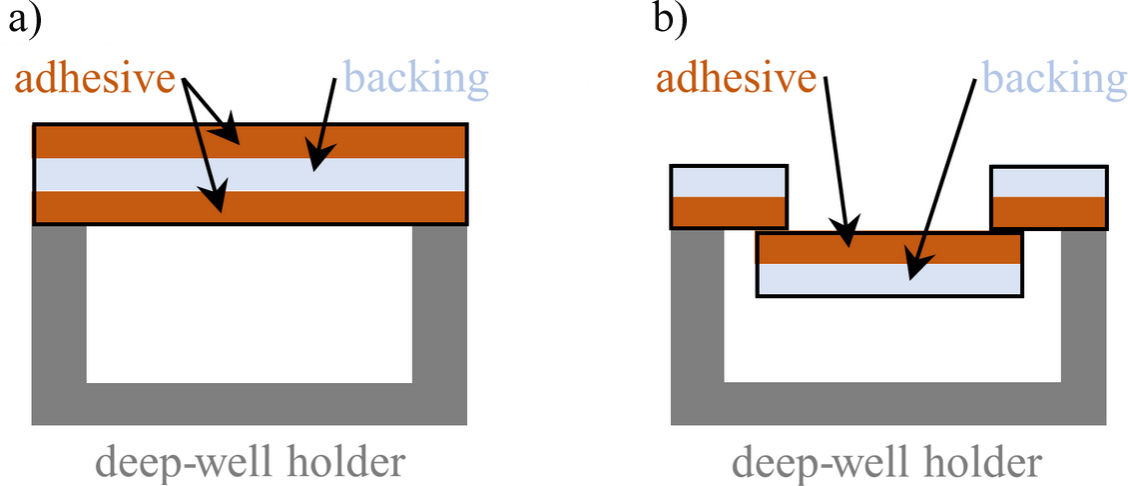
Illustrated cross sections of the adhesive tapes mounted on the deep-well specimen holder in the case of (*a*) double-sided adhesive tapes and (*b*) single-sided adhesive tapes.

Fig. 7 ▸ shows how the tapes were prepared for XRD analysis. The double-sided adhesive tapes were attached to a deep-well PMMA sample holder, which is not supposed to contribute to the measured signal as the PMMA is out of focus, with one adhesive side of the tape facing upwards [Fig. 7 ▸(*a*)]. This was also realized for single-sided adhesive tapes by inversely sticking together three tape pieces [Fig. 7 ▸(*b*)]. The sample holder with the adhesive tape was then mounted on the sample stage, with the tape being perpendicular to the X-ray beam direction [Fig. 11(*a*)]. As the common office tape width is 19 mm, tape pieces of ∼19 mm width were cut from the wider packing tapes (samples 6 and 11) before they were placed on the deep-well sample holder. Tapes with smaller widths than 19 mm (samples 4, 12 and 13) were tested as provided.

An empty deep-well holder was analysed to confirm the absence of a contribution to the measured signal when testing the adhesive tapes. For the sake of comparison, a zero-background plate, a 150 µm thick microscope glass slide, a 125 µm thick Kapton (polyimide) foil, a zero-background plate with petroleum jelly (Molyduval, pharmaceutical Vaseline) and an actual powder sample (0.02 g T6Al4V powder) prepared with the top dusted adhesive tape method were also analysed.

A Bruker D8 Discover diffractometer with a Bragg–Brentano setup [Fig. 1 ▸(*a*)], monochromatic Cu *K*α_1_ radiation and a 1D LYNXEYE detector was used for all diffraction experiments. All measurements were performed from 5° to 120° 2θ, with a step size of 0.014° and a time per step of 0.5 s. The scans were performed in coupled θ/2θ mode at a divergence slit opening degree of 0.2° without sample rotation. The omission of sample rotation is due to two technical reasons arising from the specific setup presented. Firstly, measurements starting from 5° 2θ in the coupled θ/2θ mode, which are important to reveal information about the different diffraction behaviours of the adhesive types, are only possible if the sample holder is aligned with the beam direction like in Fig. 4 ▸(*a*). Upon rotation, the mechanical stop of the sample stage [Fig. 1 ▸(*b*)] would cause a rotation-angle-dependent shadow on the sample at low X-ray incident angles. Secondly, the fixed width of the X-ray illuminated area for this setup of approximately 20 mm is larger than the typical adhesive tape width. If the adhesive tapes were not oriented perpendicular to the X-ray beam permanently, the actual area probed by the X-rays would be rotation angle dependent. If such technical restrictions do not apply, it is recommended to use sample rotation to have better statistics and to account for a potential preferred orientation of the adhesive tapes as a result of the manufacturing process of the polymer backing.

## 3. Results

**Figure 8:**
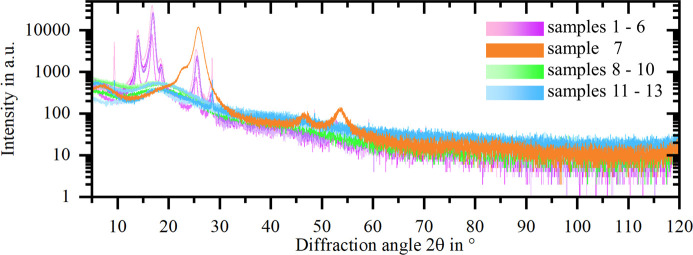
Diffractograms of all 13 tested adhesive tapes, colour-grouped according to the backing material.

Fig. 8 ▸ provides the diffractograms of all 13 adhesive tapes in a single plot. Despite identical measurement settings, the maximum obtained intensities differ by several orders of magnitude between the different adhesive tapes. The presence and position of characteristic reflections, including the corresponding peak shape and width, also differ. These differences are especially pronounced in the lower 2θ range. The colour scheme used in Fig. 8 ▸ groups the diffractograms according to the adhesive tape backing material, revealing qualitatively similar diffractograms in the case of identical backing material.

**Figure 9:**
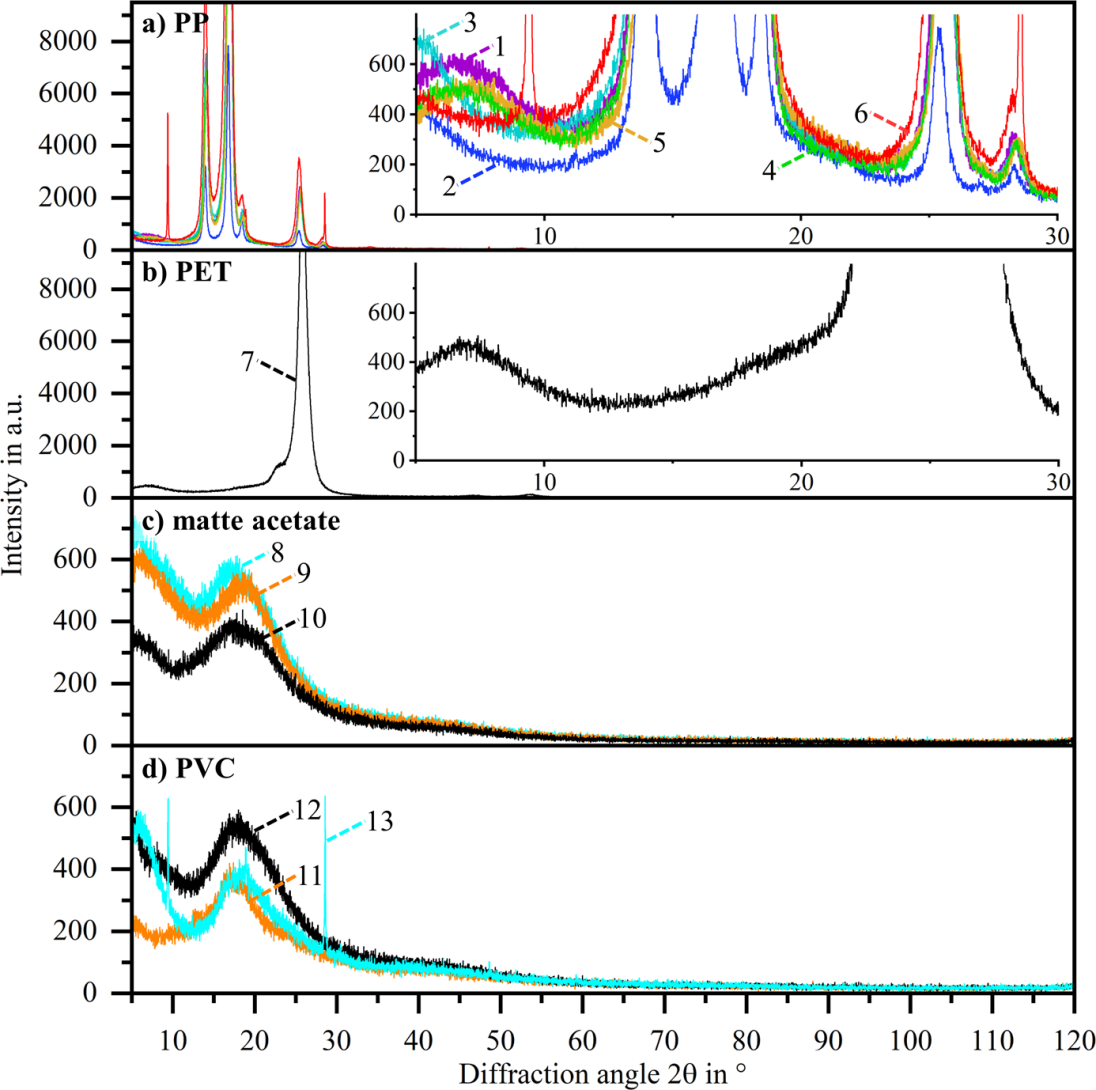
Detailed diffractograms of all 13 tested adhesive film tapes, grouped according to their backing material: (*a*) PP, (*b*) PET, (*c*) matte acetate and (*d*) PVC.

Fig. 9 ▸ depicts the diffractograms, grouped according to their backing material type, in more detail. Fig. 9 ▸(*a*) shows the diffractograms of adhesive tapes with polypropyl­ene (PP) backings. They all exhibit reflections at 14°, 17°, 18.5° and 25.5°, belonging to the monoclinic α-form of PP (Machado *et al.*, 2005 ▸). Only for sample 6 are additional distinctive reflections not belonging to α-PP apparent at 9.5° and 28.6°. Sample 6 exhibits the overall highest reflection intensities, probably related to the greater tape thickness compared with the other samples. For samples 1, 3, 4 and 5, the α-PP reflection intensities and the corresponding peak shapes are similar. When comparing the diffractograms in the lower-angle range, it is apparent that samples 1, 4 and 5 (tesa, water-based acrylic adhesive) exhibit an amorphous peak at approximately 7°, which is not present for samples 2 and 3 (Scotch, synthetic acrylic adhesive) and sample 6 (Scotch, synthetic rubber adhesive).

Fig. 9 ▸(*b*) shows the diffractogram of the adhesive tape with recycled PET backing with an amorphous peak at approximately 7°, as again a water-based acrylic adhesive is used according to the data sheet. The main reflection at approximately 26° corresponds to triclinic PET (Wakelyn, 1983 ▸), while additional reflections reported for PET films and PET fibres cannot be resolved from the peak shoulder on the lower-angle side of the main reflection.

Fig. 9 ▸(*c*) shows that the diffractograms of adhesive tapes with a matte acetate film backing, samples 8, 9 and 10 (Scotch, synthetic acrylic adhesive), are comparable. As for the PP samples 2 and 3 (also Scotch, synthetic acrylic adhesive), the measured intensity decreases in the range of 5° to 10° 2θ. A reflection of cellulose acetate at approximately 9° (Wu *et al.*, 2014 ▸) is probably convoluted with the signal originating from the adhesive, while a broad amorphous peak around 18° associated with cellulose acetate (Wu *et al.*, 2014 ▸) is visible.

Fig. 9 ▸(*d*) shows the diffractograms of the adhesive tapes with polyvinyl chloride (PVC) backing, which are characterized by a broad amorphous peak in the range of approximately 15° to 30°. The main reflections of PVC at 17.7°, 19.5°, 25.5° and 41.4° according to the literature (Edraki *et al.*, 2021 ▸) can hardly be distinguished. Sample 13 has a qualitatively different diffraction behaviour in the lower-angle range. Additional distinct reflections occur, indicating another crystalline component similar to sample 6.

**Figure 10:**
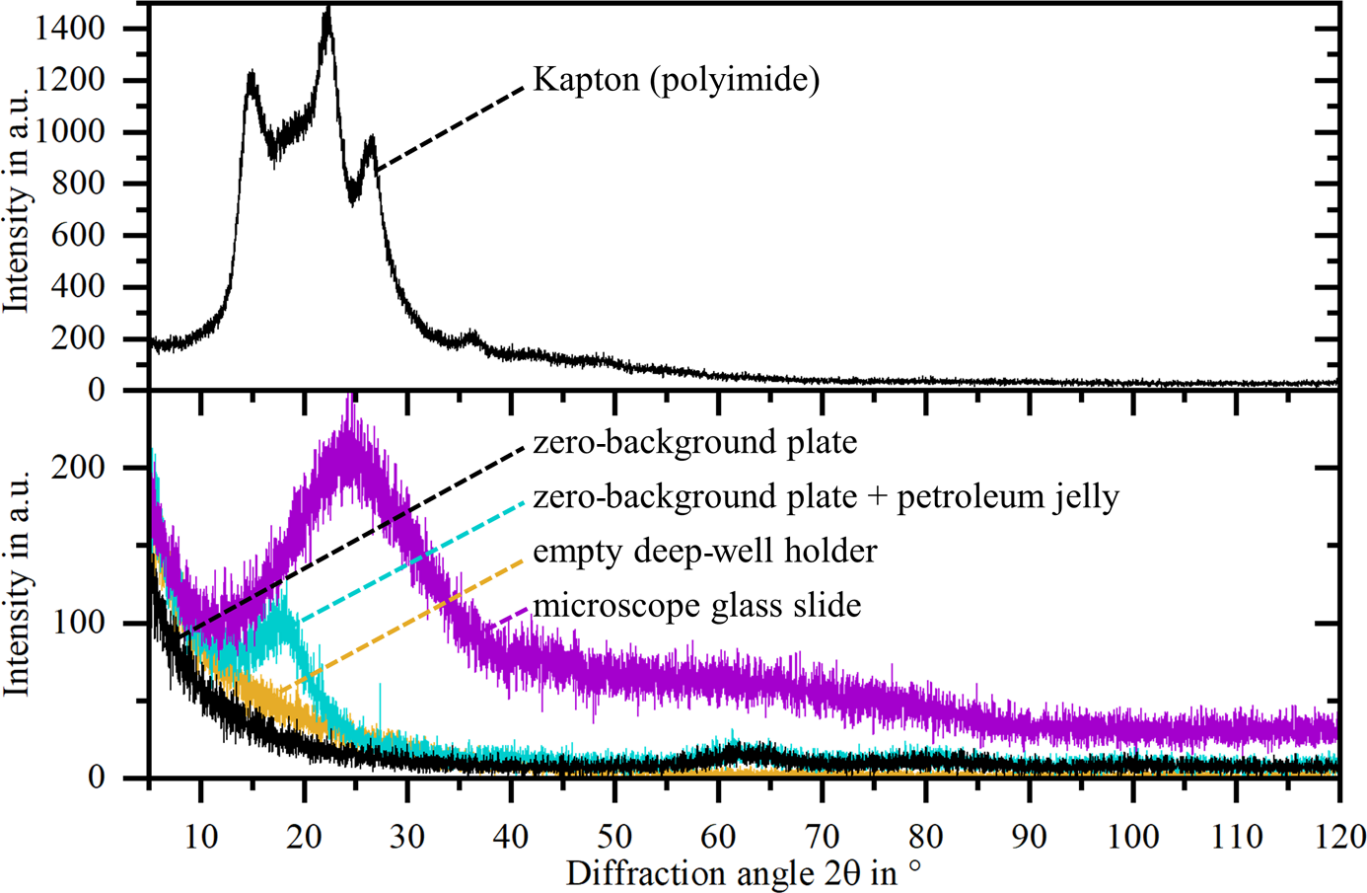
Diffractograms of a zero-background plate with and without petroleum jelly, a microscope glass slide, a Kapton foil, and an empty deep-well sample holder.

Fig. 10 ▸ provides the diffractograms of a zero-background plate with and without petroleum jelly, a microscope glass plate, a Kapton foil, and an empty deep-well sample holder. It is apparent that the empty deep-well holder does not cause any reflections or amorphous peaks. The deep-well holder is therefore a suitable carrier to mount and test the adhesive tapes with the present sample stage. The diffractogram of the zero-background plate is similar, despite a slight intensity increase between 55° and 90°. Adding petroleum jelly to the zero-background plate as adhesion promoter causes an amorphous peak to occur at approximately 18° 2θ. Despite the selected petroleum jelly being pharmaceutical Vaseline, tiny additional reflections, *e.g.* at approximately 21.4° 2θ, can be identified due to potential impurities or additives, and these have to be considered. The microscope glass slide exhibits a broad amorphous peak at 25° even without petroleum jelly. Note that, at identical measurement settings, the obtained maximum intensities for these substrates remain below those observed for adhesive tapes. By contrast, Kapton leads to several distinct reflections of significantly higher intensity, matching published diffractograms [*e.g.* Cardoso *et al.* (2001 ▸)].

**Figure 11:**
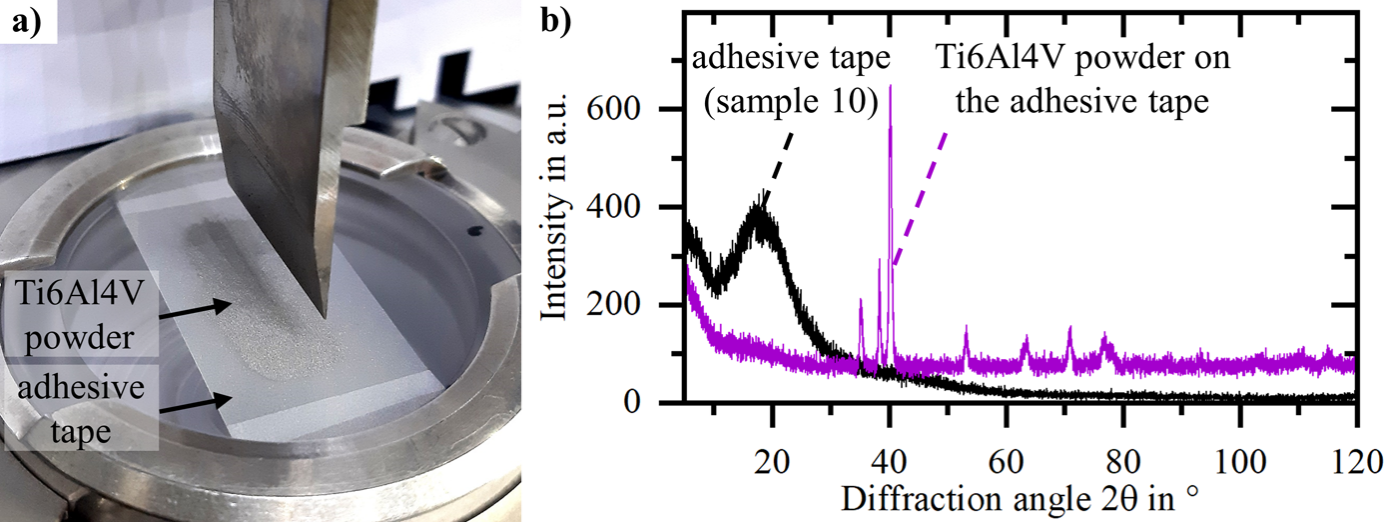
(*a*) Practical implementation of the top dusted adhesive tape method using Ti6Al4V powder on a matte acetate adhesive tape (sample 10) with (*b*) the corresponding diffractograms.

Fig. 11 ▸(*a*) presents an actual implementation of the top dusted adhesive tape method. A sample of 0.02 g of Ti6Al4V powder dusted onto a matte acetate adhesive tape (sample 10) is sufficient to largely cover the X-ray irradiated area of this setup. Due to the low-intensity background caused by the chosen matte acetate adhesive film, the reflections belonging to the Ti6Al4V powder sample can be clearly identified [Fig. 11 ▸(*b*)]. The amorphous peak around 18° observed for the matte acetate adhesive film is significantly reduced due to the presence of the Ti6Al4V powder covering the adhesive film, reducing the tape’s contribution to the measured signal. At this point it has to be mentioned that the actual contribution of the adhesive tape to the measured signal depends on how well the powder sample covers the adhesive tape and how X-ray transparent the sample is. Furthermore, the X-ray penetration depth in the Bragg–Brentano setup is dependent on the incident angle, meaning that at larger incident angles the X-rays have a higher penetration depth (Harrington & Santiso, 2021 ▸) and hence are more likely to probe the adhesive tape.

## 4. Discussion

The diffraction behaviour of commercially available adhesive tapes differs significantly depending on the backing material and the adhesive type used. The backing material is decisive if one or more intense characteristic reflections (PP, PET) or a few amorphous peaks (matte acetate, PVC) occur. Depending on the adhesive type, amorphous peaks occur especially in the lower-2θ range. Additional crystalline reflections can occur.

Given the nature of the top dusted adhesive tape method, X-rays interact not only with the sample powder but also with the adhesive tape. This results in an adhesive-tape-specific background noise. A common goal in specimen preparation is to eliminate or minimize such background (Buhrke *et al.*, 1999 ▸). In this respect, this study outlines that certain tapes are better suited to the top dusted adhesive tape sample preparation method than others. Aiming for a low background and an absence of high-intensity reflections over a large 2θ range, tapes with matte acetate backing, like sample 10 (Scotch, ‘Removable Invisible Tape 811’), or with a PVC backing, like sample 11 (tesa, ‘tesapack ultra strong transparent’), are a good choice.

Some PP and PET tapes are only an option for powder samples where the range of interest is very narrow and at lower angles. They provide small windows of near-constant background, *e.g.* sample 2 (PP) between 7.5° and 12.5° or sample 7 (PET) between 10° and 15°. The ‘ideal’ tape is thus dependent on the requirements defined by the user with regard to the powder sample to be measured.

A direct comparison of the top dusted adhesive tape method with the conventional top dusted method reveals that all 13 adhesive tapes lead to a higher undesired background than a zero-background plate. The microscope glass slide also performs better than the adhesive tapes. On the other hand, Kapton, which is often used in XRD applications, leads to a significant background with distinct reflections. Petroleum jelly as adhesion promoter adds only a comparatively small amorphous peak to the background. If the lowest possible background signal is a priority, using the top dusted method with a zero-background plate as carrier and petroleum jelly as adhesion promoter is preferable. This setup also has a better positioning accuracy because, unlike adhesive tapes, the carrier material is not intrinsically flexible and subject to bending or wrinkling.

However, the top dusted adhesive tape method has other advantages. Adhesive tapes are very cheap and easily available in most laboratories. Hence, several specimens can be prepared in a row without the need to reuse the same zero-background plate. This saves time and costs, as preparation of the zero-background plate with petroleum jelly and a cleaning procedure after the measurements are omitted. Furthermore, such a cleaning procedure has the disadvantage that the powder sample is lost for further analysis. In contrast, once measured, the dusted adhesive tapes can be stored or archived and remeasured at a later point. An additional advantage when using adhesive film tapes is that the thickness of the adhesive layer is always constant. This is not the case for petroleum jelly manually applied on a carrier, which causes the amorphous peak associated with the petroleum jelly to vary between different experiments.

## 5. Conclusion

The top dusted adhesive tape sample preparation method is an inexpensive and attractive option for the XRD analysis of small powder volumes. Unlike the conventional top dusted method, typically requiring a zero-background sample holder as carrier plate, the powder of interest is dusted onto an adhesive tape.

The diffractograms of commercially available adhesive tapes published in this work will assist XRD users in choosing the best and most suitable adhesive tape for their purpose. In general, tapes with a matte acetate or PVC backing are recommended since they cause only an overall low background signal without high-intensity reflections.

While a background as low as that of a zero-background specimen holder cannot be achieved, adhesive tapes are outstanding regarding their cost, availability and ease of use, the option of storing and remeasuring multiple specimens, and the absence of cleaning issues.

## Supplementary Material

Original experimental diffractograms of all tested adhesive tapes: https://doi.org/10.17632/h6b36vt4st.2

## Figures and Tables

**Table 1:** Overview of the tested commercial adhesive tapes N.A. indicates that the information is not available.

ID	Brand	Product name	Company product No.	International article No. (EAN)	Backing material	Adhesive type (* if double-sided tape)	Total thickness (µm)
1	tesa	tesafilm crystal clear	57330	4042448040039	PP (biaxially oriented)	Water-based acrylic	55
2	Scotch	Crystal Clear Tape 600	7100027387	3134375261920	PP	Synthetic acrylic	51
3	Scotch	Transparent Tape 550	7100194348	3134375305150	PP	Synthetic acrylic	55
4	tesa	tesafilm standard	57206	4042448049698	PP (biaxially oriented)	Water-based acrylic	44
5	tesa	tesafilm invisible	57312	4042448039910	PP (biaxially oriented)	Water-based acrylic	52
6	Scotch	BoxLock Packing Tape	7100263253	0638060856420	PP	Synthetic rubber	78.7 (backing)
7	tesa	tesafilm Eco & Crystal	59032	4063565252259	PET (90% recycled)	Water-based acrylic	44
8	Scotch	Magic Tape 810	7100069922	3134375267304	Matte acetate	Synthetic acrylic	60
9	Scotch	Magic Tape 900	7100044084	0051141405995	Matte acetate	Synthetic acrylic	N.A.
10	Scotch	Removable Invisible Tape 811	7000029163	021200728235	Matte acetate	Synthetic acrylic	50
11	tesa	tesapack ultra strong transparent	57176	4042448123619	PVC	Natural rubber	65
12	Scotch	Double-Sided Tape 136D-MDOEU	7100276528	051131598546	PVC (un-plasticized)	*Synthetic acrylic	N.A.
13	tesa	tesa Photo Film	56661	4042448030733	PVC (un-plasticized)	*Polyacrylate	N.A.

## Data Availability

The original experimental data are freely accessible from Mendeley Data, https://doi.org/10.17632/h6b36vt4st.2.
